# Linear association between serum potassium levels and 28-day mortality among ICU patients with diabetes and sepsis: a multicenter study

**DOI:** 10.3389/fmed.2025.1582894

**Published:** 2025-05-30

**Authors:** Anxiang Cai, Tianyi Zhang, Kaiwen Gao, Xinglin Chen, Shu Li, Qisheng Lin, Shan Mou, Zhaohui Ni, Haijiao Jin

**Affiliations:** ^1^Department of Nephrology, Ren Ji Hospital, Shanghai Jiao Tong University School of Medicine, Shanghai, China; ^2^Molecular Cell Laboratory for Kidney Disease, Ren Ji Hospital, Shanghai Jiao Tong University School of Medicine, Shanghai, China; ^3^Shanghai Peritoneal Dialysis Research Center, Ren Ji Hospital, Shanghai Jiao Tong University School of Medicine, Shanghai, China; ^4^Uremia Diagnosis and Treatment Center, Shanghai Jiao Tong University School of Medicine, Shanghai, China; ^5^Department of Critical Care Medicine, Ren Ji Hospital, Shanghai Jiao Tong University School of Medicine, Shanghai, China; ^6^Department of Epidemiology and Biostatistics, Empower U, X&Y Solutions Inc., Boston, MA, United States

**Keywords:** serum potassium, diabetes, sepsis, ICU, mortality, hyperkalemia, hypokalemia

## Abstract

**Background:**

Dysregulation of serum potassium is a common electrolyte disturbance in critically ill patients, and both hypokalemia and hyperkalemia have been linked to adverse outcomes in sepsis. However, the relationship between serum potassium levels and mortality in ICU patients with diabetes and sepsis remains poorly understood.

**Methods:**

A retrospective cohort study was conducted using data from the eICU Collaborative Research Database (2014–2015). The study included 5,104 adult ICU patients with diabetes and sepsis from 208 hospitals in the U.S. Serum potassium levels measured within 24 h of ICU admission were categorized into hypokalemia (<3.5 mmol/L), normokalemia (3.5–5.0 mmol/L), and hyperkalemia (>5.0 mmol/L). Multivariable logistic regression models were used to assess the association between serum potassium levels and 28-day ICU mortality.

**Results:**

Of the 5,104 patients (mean age, 66.8 years; 49.1% male), 1,046 (20.5%) had hypokalemia, 3,377 (66.2%) had normokalemia, and 681 (13.3%) had hyperkalemia. After adjusting for demographic factors, comorbidities, and treatment measures, each 1 mmol/L increase in serum potassium was associated with a 25% higher risk of 28-day mortality (adjusted OR, 1.25; 95% CI, 1.07–1.47). Compared to hypokalemia, hyperkalemia was associated with significantly higher mortality risk (adjusted OR, 1.86; 95% CI, 1.17–2.96). A linear relationship was observed between serum potassium levels and mortality (*P* = 0.006), differing from the previously reported U-shaped relationship in general ICU populations.

**Conclusions and relevance:**

Elevated serum potassium levels were independently associated with increased 28-day mortality in ICU patients with diabetes and sepsis. These findings suggest that potassium management strategies should be specifically tailored for this high-risk patient population.

## Introduction

Sepsis remains a major global health challenge, affecting millions of patients annually and carrying substantial mortality rates, particularly among those requiring intensive care unit (ICU) admission. Despite advances in critical care medicine, the mortality rate for patients with sepsis in ICUs ranges from 25 to 50%, highlighting the urgent need to identify modifiable risk factors that could improve outcomes (1, 2).

Dysregulation of serum potassium, a crucial electrolyte for maintaining cellular function and cardiovascular stability, has been associated with increased mortality risk in critically ill patients (3, 4). Several studies have suggested a U-shaped relationship between serum potassium levels and mortality in general and septic ICU populations, with both hypokalemia and hyperkalemia linked to adverse outcomes (5–9). However, this relationship may not be consistent across all patient subgroups. Recent evidence suggests that the association between serum potassium and mortality might vary depending on underlying comorbidities and patient characteristics (10, 11).

Diabetes mellitus, affecting ~20–35% of ICU patients with sepsis, has been identified as a significant risk factor for potassium homeostasis disorders (12–14). Previous research demonstrated that diabetes is independently associated with an increased risk of hyperkalemia, potentially due to insulin resistance, impaired potassium cellular uptake, and diabetic kidney disease (15–18). However, the relationship between serum potassium levels and mortality specifically in diabetic patients with sepsis remains poorly understood, as most existing studies have either excluded this population or included it as part of general ICU cohorts (5–8, 19–21).

Therefore, we conducted this study to investigate the association between serum potassium levels and 28-day mortality in ICU patients with both sepsis and diabetes, aiming to better understand whether the traditional U-shaped relationship between potassium and mortality holds true in this specific population.

## Methods

### Data source and population

The study participants were identified from the eICU Collaborative Research Database (version 2.0) (22), a multicenter database comprising the data of patients admitted to the intensive care unit (ICU) in the United States (US). This database contains high-granularity medical data from 200,859 admissions to the ICUs across 208 hospitals during 2014–2015 and is accessible to the public. The eICU Collaborative Research Database includes diverse clinical data, such as information on demographic characteristics, vital signs, laboratory tests, disease severity measures, diagnosis, and treatment approaches. The data were collected and normalized based on an effective electronic clinical management system. One of our authors was responsible for data extraction after gaining access to the database.

**Figure 1:**
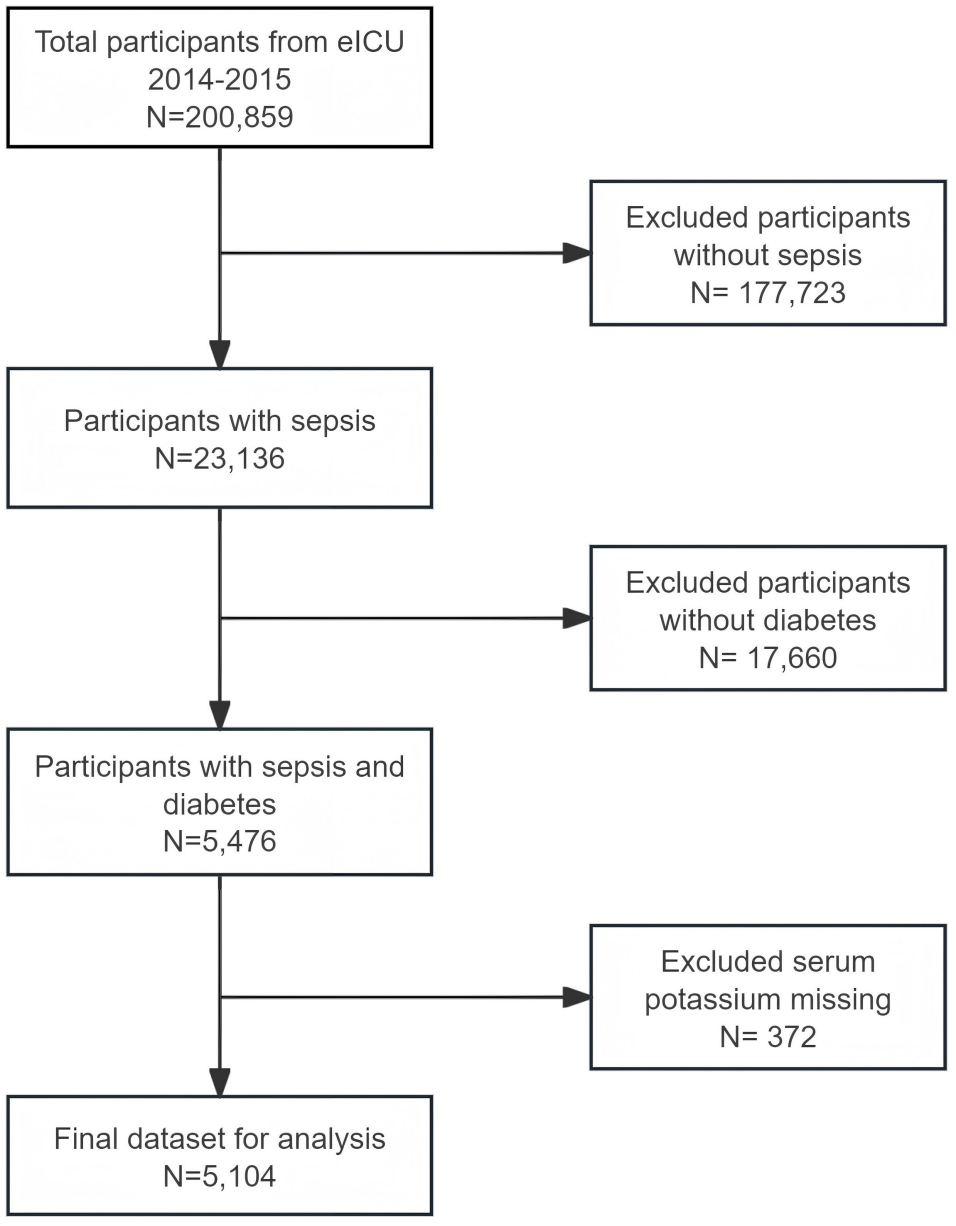
Flow chart of participants selection.

In this study, all patients diagnosed with sepsis and diabetes were considered for inclusion. We excluded patients with missing values of serum potassium within 24 h of admission. Ultimately, 5,104 eligible participants were included in our final analysis (Figure 1). Serum potassium levels measured within 24 h of ICU admission were categorized into hypokalemia (<3.5 mmol/L), normokalemia (3.5–5.0 mmol/L), and hyperkalemia (>5.0 mmol/L), consistent with commonly used clinical thresholds.

### Variable extraction

Baseline characteristics of patients, including demographic data, comorbidities, source of infection, clinical characteristics, laboratory values, and treatment strategies, within 24 h of ICU admission were extracted to avoid potential confounders. Demographics included age, sex and ethnicity. Comorbidities included acute kidney injury (AKI), acute myocardial infarction (AMI), congestive heart failure (CHF), cardiac arrhythmia, pneumonia, chronic obstructive pulmonary disease (COPD), cirrhosis, metastatic cancer, lymphoma, leukemia and immunosuppression. Source of infection included pulmonary, renal/urinary tract, gastrointestinal, cutaneous/soft tissue, gynecologic, other or unknown infection. Clinical characteristics included body mass index (BMI) and sequential organ failure assessment (SOFA) score. Laboratory parameters included serum potassium, serum creatinine, blood urea nitrogen (BUN), glucose, serum sodium, serum chloride, ionized calcium, serum albumin, serum prealbumin, 24 h urine protein, hemoglobin, platelet count, erythrocyte sedimentation rate (ESR), C-reactive protein (CRP), Troponin-I, lactate dehydrogenase (LDH), creatine phosphokinase-myocardial band (CPK-MB), creatine phosphokinase (CPK), low-density lipoprotein cholesterol (LDLc), total cholesterol, triglycerides, high-density lipoprotein cholesterol (HDLc), uric acid, lipase and amylase. Regarding treatment measures, the usage of mechanical ventilation, dialysis, and vasopressor were included.

### Outcomes

Primary outcome were defined as all-cause 28-day mortality in ICU.

### Statistical analysis

Depending on whether or not it conformed to a normal distribution, continuous variables were presented as mean (standard deviation). Categorical variables were described as frequencies (percentages). The differences among individuals were assessed using the Kruskal-Wallis rank-sum test or Fisher's exact test. A two-sided *P*-value <0.05 was considered statistically significant.

To investigate the functional form of the relationship between serum potassium levels and 28-day ICU mortality, we conducted a smooth curve fitting analysis using generalized additive models with smoothing splines. In this analysis, we adjusted for age, sex, ethnicity, comorbidities (AKI, CHF, metastatic cancer), BMI, SOFA score, laboratory values (serum creatinine, BUN, glucose, serum sodium, serum chloride, serum albumin, and platelet count) and treatment measures (use of mechanical ventilation and dialysis).

After examining the relationship pattern, we used logistic regression models to quantify the association between serum potassium and 28-day ICU mortality. Data were presented as odds ratios (OR) with 95% confidence intervals (CI) to represent the effect of serum potassium on 28-day ICU mortality risk. For multivariable models, we included factors previously demonstrated to be prognostically significant, variables considered clinically important, and covariates identified in univariate logistic regression as significant predictors of mortality. We constructed the following sequential models to determine the influence of potential confounders on the serum potassium-mortality relationship: unadjusted; model 1, adjusted only for age and sex; model 2, adjusted for age, sex, and serum creatinine; and model 3, fully adjusted for age, sex, ethnicity, comorbidities (AKI, CHF, metastatic cancer), BMI, SOFA score, laboratory values (serum creatinine, BUN, glucose, serum sodium, serum chloride, serum albumin, and platelet count), and treatment measures (mechanical ventilation and dialysis). The covariates in the fully adjusted model were consistent with those used in the previous smooth curve fitting analysis. In all models, 28-day ICU mortality was the dependent variable, while serum potassium was analyzed as a continuous variable, as categorical variables based on clinical thresholds (<3.5, 3.5–5.0, >5.0 mmol/L), and as ordinal categories to assess for potential dose-response relationships.

To explore potential effect modification, we established subgroup-stratified models examining the association between serum potassium and 28-day ICU mortality across different patient characteristics, including age, sex, comorbidities (AKI, CHF, metastatic cancer), clinical parameters (BMI, SOFA score), laboratory values (serum creatinine, BUN, glucose, serum sodium, serum chloride, serum albumin, platelets), and treatment measures (mechanical ventilation and dialysis).

The statistical analysis was conducted using EmpowerStats (V4.2, https://www.empowerstats.net/en/) and R (V3.4.3, http://www.R-project.org).

## Results

### Patient characteristics and clinical features

In this cohort of 5,104 ICU patients with diabetes and sepsis (mean age, 66.8 years; 49.1% male), patients were stratified into three groups based on serum potassium levels: hypokalemia (<3.5 mmol/L, *n* = 1,046), normokalemia (3.5–5.0 mmol/L, *n* = 3,377), and hyperkalemia (>5.0 mmol/L, *n* = 681). Significant differences were observed in age (65.0 ± 13.9 vs. 67.4 ± 13.1 vs. 66.5 ± 13.1 years, *P* < 0.001) and sex distribution (male: 57.4% vs. 47.2% vs. 45.5%, *P* < 0.001) among the groups. Regarding comorbidities, AKI (38.5% vs. 23.5% vs. 20.4%, *P* < 0.001) and CHF (11.7% vs. 8.4% vs. 8.0%, *P* = 0.011) were more prevalent in the hyperkalemia group, while metastatic cancer showed lower prevalence (3.2% vs. 2.7% vs. 4.3%, *P* = 0.031). The hyperkalemia group demonstrated higher BMI (34.2 ± 11.2 vs. 32.0 ± 9.8 vs. 30.7 ± 9.3, *P* < 0.001) and more severe organ dysfunction (SOFA score: 5.7 ± 2.9 vs. 4.3 ± 2.9 vs. 4.1 ± 2.8, *P* < 0.001). Laboratory findings revealed significantly impaired renal function in the hyperkalemia group, with elevated serum creatinine (3.6 ± 2.5 vs. 2.1 ± 1.9 vs. 1.7 ± 1.5 mg/dL, *P* < 0.001) and BUN (58.6 ± 30.9 vs. 37.6 ± 24.7 vs. 30.0 ± 21.1 mg/dL, *P* < 0.001). Additionally, the hyperkalemia group showed higher glucose levels (203.0 ± 138.9 vs. 185.4 ± 112.6 vs. 180.3 ± 112.6 mg/dL, *P* < 0.001) and lower serum sodium (136.3 ± 6.3 vs. 137.9 ± 5.7 vs. 139.5 ± 6.7 mmol/L, *P* < 0.001) and serum chloride levels (102.9 ± 7.7 vs. 104.3 ± 6.9 vs. 105.3 ± 8.0 mmol/L, *P* < 0.001). Higher platelet count (215.5 ± 119.8 vs. 206.7 ± 109.7 vs. 198.2 ± 107.2 × 10^9^/L, *P* = 0.008) were also observed in the hyperkalemia group. Regarding therapeutic interventions, the hyperkalemia group had higher rates of mechanical ventilation (35.1% vs. 24.8% vs. 28.5%, *P* < 0.001) and dialysis (15.0% vs. 10.2% vs. 6.9%, *P* < 0.001). Notably, the 28-day ICU mortality was significantly higher in the hyperkalemia group (14.4% vs. 7.1% vs. 6.2%, *P* < 0.001; Table 1).

**Table 1:** Baseline characteristics of ICU patients with diabetes and sepsis by serum potassium levels.

**Characteristic**	**Serum potassium level**			***P*-value**
	<**3.5 mmol/L (*****n*** = **1,046)**	**3.5–5.0 mmol/L (*****n*** = **3,377)**	>**5.0 mmol/L (*****n*** = **681)**	
**Demographics**				
Age, mean (SD), y	65.0 (13.9)	67.4 (13.1)	66.5 (13.1)	<0.001
Male sex, no. (%)	600 (57.4)	1,595 (47.2)	310 (45.5)	<0.001
Ethnicity, no. (%)				0.220
Caucasian	772 (73.9)	2,531 (75.2)	515 (76.0)	
African American	127 (12.2)	372 (11.1)	93 (13.7)	
Hispanic	67 (6.4)	187 (5.6)	33 (4.9)	
Asian	39 (3.7)	148 (4.4)	18 (2.7)	
Native American	18 (1.7)	50 (1.5)	10 (1.5)	
Other/unknown	22 (2.1)	76 (2.3)	9 (1.3)	
**Comorbidities, no. (%)**				
AKI	213 (20.4)	793 (23.5)	262 (38.5)	<0.001
AMI	46 (4.4%)	114 (3.4%)	26 (3.8%)	0.295
Congestive heart failure	84 (8.0)	282 (8.4)	80 (11.7)	0.011
Cardiac arrhythmia	136 (13.0%)	444 (13.1%)	91 (13.4%)	0.977
Pneumonia	286 (27.3%)	953 (28.2%)	184 (27.0%)	0.742
COPD	63 (6.0)	256 (7.6)	51 (7.5)	0.229
Cirrhosis	29 (2.8)	113 (3.3)	23 (3.4)	0.640
Metastatic cancer	45 (4.3)	91 (2.7)	22 (3.2)	0.031
Lymphoma	15 (1.4%)	32 (0.9%)	4 (0.6%)	0.196
Leukemia	18 (1.7%)	44 (1.3%)	11 (1.6%)	0.554
Immunosuppression	62 (5.9%)	171 (5.1%)	37 (5.4%)	0.543
**Source of infection, no. (%)**				0.069
Pulmonary	338 (32.3)	1,180 (34.9)	221 (32.5)	
Renal/UTI	316 (30.2)	857 (25.4)	177 (26.0)	
Gastrointestinal	107 (10.2)	341 (10.1)	77 (11.3)	
Cutaneous/soft tissue	102 (9.8)	397 (11.8)	87 (12.8)	
gynecologic	4 (0.4)	7 (0.2)	2 (0.3)	
Other	70 (6.7)	182 (5.4)	36 (5.3)	
Unknown	109 (10.4)	413 (12.2)	81 (11.9)	
**Clinical characteristics, mean (SD)**				
BMI, kg/m^2^	30.7 (9.3)	32.0 (9.8)	34.2 (11.2)	<0.001
SOFA score	4.1 (2.8)	4.3 (2.9)	5.7 (2.9)	<0.001
**Laboratory values, mean (SD)**				
Serum potassium, mmol/L	3.2 ± 0.3	4.2 ± 0.4	5.7 ± 0.6	<0.001
Serum creatinine, mg/dL	1.7 (1.5)	2.1 (1.9)	3.6 (2.5)	<0.001
Blood urea nitrogen, mg/dL	30.0 (21.1)	37.6 (24.7)	58.6 (30.9)	<0.001
Glucose, mg/dL	180.3 (112.6)	185.4 (112.6)	203.0 (138.9)	<0.001
Serum sodium, mmol/L	139.5 (6.7)	137.9 (5.7)	136.3 (6.3)	<0.001
Serum chloride, mmol/L	105.3 (8.0)	104.3 (6.9)	102.9 (7.7)	<0.001
Ionized calcium, mmol/L	3.8 ± 1.3	3.9 ± 1.4	3.8 ± 1.3	0.534
Serum albumin, g/dL	2.5 (0.6)	2.6 (0.6)	2.6 (0.6)	<0.001
Serum prealbumin, mg/dL	10.7 ± 6.0	11.1 ± 7.7	10.0 ± 5.8	0.873
24 h urine protein, mg/24 h	60.0 ± 37.4	210.4 ± 795.2	386.6 ± 485.3	0.752
Hemoglobin, g/dL	10.2 ± 2.0	10.2 ± 2.0	10.1 ± 2.3	0.248
Platelets, cells × 10^9^/L	198.2 ± 107.2	206.7 ± 109.7	215.5 ± 119.8	0.008
ESR, mm/h	48.1 ± 38.1	55.1 ± 34.0	61.2 ± 41.2	0.428
CRP, mg/dL	317.8 ± 747.0	219.4 ± 583.8	144.9 ± 518.8	0.573
Troponin—I, ng/mL	2.0 ± 7.5	2.0 ± 8.1	1.5 ± 4.2	0.694
LDH, Units/L	664.9 ± 1,356.2	605.8 ± 918.1	508.4 ± 709.9	0.844
CPK-MB, ng/mL	10.5 ± 20.4	12.2 ± 26.8	11.7 ± 18.8	0.819
CPK, Units/L	1,178.4 ± 5,941.7	1,255.6 ± 11,471.2	825.5 ± 2,133.9	0.878
LDLc, mg/dL	56.0 ± 29.8	53.7 ± 29.8	48.4 ± 21.7	0.535
Total cholesterol, mg/dL	119.5 ± 48.8	116.5 ± 40.5	107.0 ± 38.0	0.410
Triglycerides, mg/dL	157.9 ± 119.4	162.5 ± 114.2	266.7 ± 720.4	0.055
HDLc, mg/dL	34.1 ± 15.6	31.0 ± 13.2	29.0 ± 14.0	0.154
Uric acid, mg/dL	7.9 ± 2.9	7.6 ± 3.2	8.7 ± 2.9	0.286
Lipase, Units/L	236.2 ± 565.3	364.5 ± 1,061.8	304.2 ± 914.4	0.602
Amylase, Units/L	174.6 ± 501.4	133.3 ± 273.0	126.8 ± 221.9	0.758
**Treatment measures, no. (%)**				
Mechanical ventilation	298 (28.5)	839 (24.8)	239 (35.1)	<0.001
Dialysis	72 (6.9)	346 (10.2)	102 (15.0)	<0.001
Vasopressor use	4 (0.4)	15 (0.4)	5 (0.7)	0.537
**Primary outcome**				
ICU 28-day mortality	65 (6.2)	241 (7.1)	98 (14.4)	<0.001

ICU, intensive care unit; AKI, acute kidney injury; AMI, acute myocardial infarction; COPD, chronic obstructive pulmonary disease; UTI, urinary tract infection; BMI, body mass index; SOFA, Sequential Organ Failure Assessment; ESR, erythrocyte sedimentation rate; CRP, C-reactive protein; LDH, lactate dehydrogenase; CPK-MB, creatine phosphokinase-myocardial band; CPK, creatine phosphokinase; LDLc, low-density lipoprotein cholesterol; HDLc, high-density lipoprotein cholesterol.

**Figure 2:**
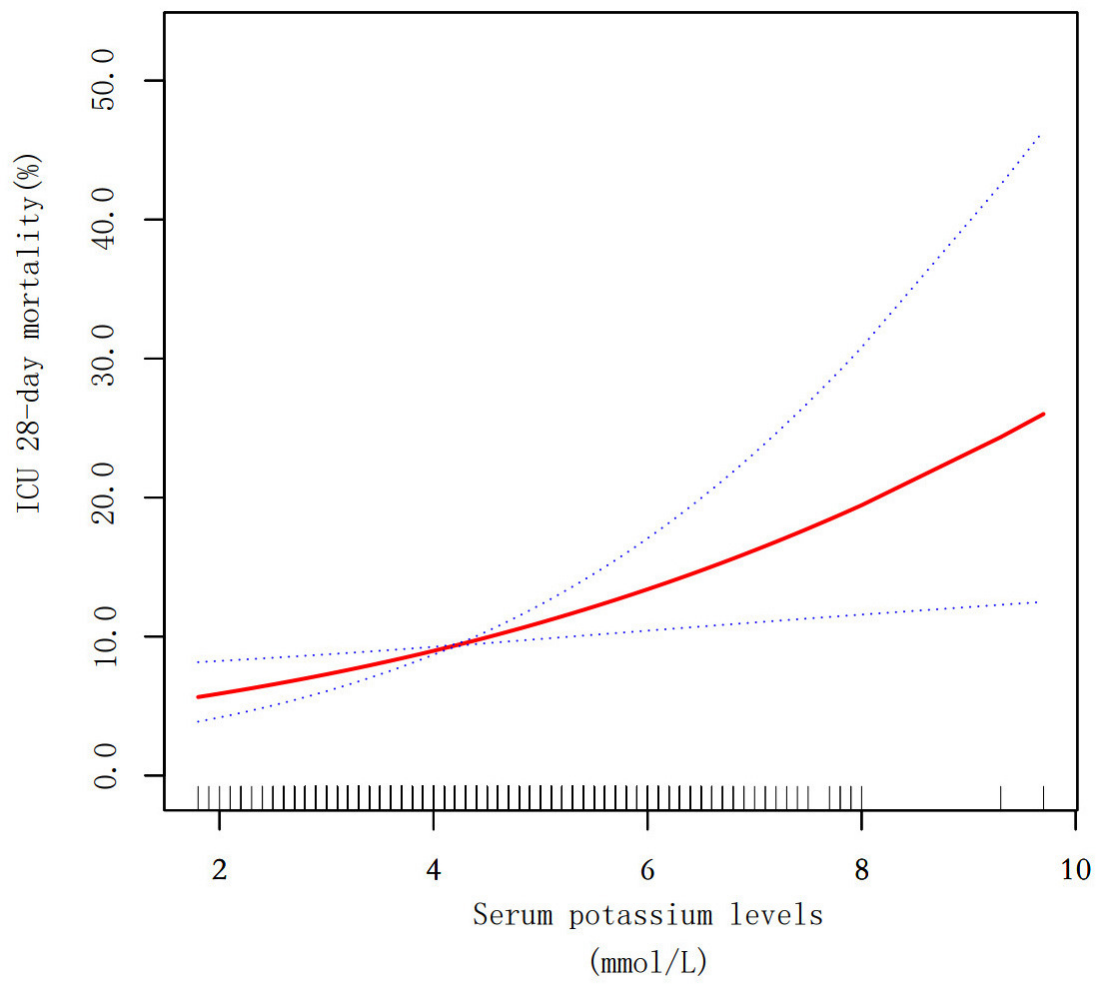
Smooth curve fitting for serum potassium and ICU 28-day mortality in ICU patients with diabetes and sepsis. The red solid line represents the fitted curve, while the blue dotted lines indicate the 95% confidence intervals. The y-axis shows mortality probability (%), and the x-axis displays serum potassium levels (mmol/L).

To explore the relationship between serum potassium levels and 28-day mortality, we conducted a smooth curve fitting analysis using generalized additive models. After fully adjustment, the results demonstrated a linear relationship between serum potassium levels and 28-day mortality (effective degrees of freedom = 1.07; *P* = 0.006; Figure 2).

In the univariate analysis, serum potassium was significantly associated with increased mortality risk (OR 1.48, 95% CI 1.32–1.66, *P* < 0.001). This association between serum potassium levels and 28-day mortality remained consistent across most prespecified subgroups. Age-stratified analysis revealed that this association strengthened with increasing age. Gender stratification showed similar association strengths for both males and females. In comorbidity subgroup analyses, the association remained significant regardless of AKI or CHF. When stratified by organ dysfunction severity, the association was most pronounced in patients with moderate SOFA scores [3–4 points: OR, 1.51 (95% CI, 1.13–2.01)] and remained significant in those with severe organ dysfunction [SOFA ≥5: OR, 1.28 (95% CI, 1.13–1.45)]. Among patients with different degrees of kidney dysfunction, the strongest association was observed in those with moderate renal impairment [serum creatinine 1.2–2.4 mg/dL: OR, 1.39 (95% CI, 1.13–1.70)]. Moreover, this association was significant among patients not receiving dialysis [OR, 1.49 (95% CI, 1.32–1.68); *P* < 0.001], while this association was not statistically significant in patients undergoing dialysis [OR, 1.32 (95% CI, 0.95–1.82); *P* = 0.094]. The relationship remained significant across various levels of other clinical parameters, including BMI, glucose, serum albumin, and platelet counts and use of mechanical ventilation status (Table 2).

**Table 2:** Stratified analysis of association between serum potassium and 28-day mortality in ICU patients with diabetes and sepsis.

**Stratification variable**	**No. of patients**	**OR (95% CI)^a^**	***P*-value**
**Demographics**			
**Age, years**			
≤ 60	1,535	1.28 (1.00–1.63)	0.050
60–70	1,444	1.33 (1.07–1.66)	0.011
>70	2,125	1.67 (1.42–1.96)	<0.001
**Sex**			
Male	2,505	1.44 (1.23–1.69)	<0.001
Female	2,597	1.53 (1.30–1.79)	<0.001
**Comorbidities**			
**Acute kidney injury**			
Yes	1,268	1.31 (1.10–1.56)	0.002
No	3,836	1.48 (1.28–1.73)	<0.001
**Congestive heart failure**			
Yes	446	1.56 (1.15–2.13)	0.004
No	4,658	1.46 (1.29–1.65)	<0.001
**Metastatic cancer**			
Yes	158	1.44 (0.79–2.61)	0.230
No	4,946	1.49 (1.33–1.67)	<0.001
**Clinical characteristics**			
**BMI, kg/m** ^2^			
<28	1,950	1.52 (1.27–1.81)	<0.001
30–34	1,326	1.75 (1.40, 2.19)	<0.001
≥34	1,663	1.28 (1.04–1.58)	0.020
**SOFA score**			
0–2	1,545	1.16 (0.69–1.94)	0.577
3–4	1,299	1.51 (1.13–2.01)	0.005
≥5	2,260	1.28 (1.13–1.45)	<0.001
**Laboratory values**			
**Serum creatinine, mg/dL**			
<1.2	1,685	1.00 (0.62–1.59)	0.984
1.2–2.4	1,825	1.39 (1.13–1.70)	0.002
≥2.4	1,555	1.19 (1.02–1.39)	0.031
**Blood urea nitrogen, mg/dL**			
<24	1,640	1.39 (0.94–2.07)	0.103
24–43	1,700	1.27 (1.01–1.60)	0.043
≥43	1,725	1.25 (1.07–1.46)	0.004
**Glucose, mg/dL**			
<129	1,650	1.59 (1.32–1.93)	<0.001
130–196	1,699	1.42 (1.14–1.77)	0.002
≥197	1,679	1.45 (1.20–1.75)	0.001
**Serum sodium, mmol/L**			
<135	1,568	1.35 (1.11–1.65)	<0.003
135–140	1,667	1.56 (1.26–1.94)	<0.001
≥140	1,844	1.59 (1.32–1.90)	<0.001
**Serum chloride, mmol/L**			
<101	1,456	1.45 (1.18–1.77)	<0.003
101–106	1,777	1.63 (1.32–2.00)	<0.001
≥107	1,835	1.39 (1.57–1.67)	0.001
**Serum albumin, g/dL**			
<2.2	883	1.45 (1.18–1.76)	<0.001
2.2–2.8	1,217	1.54 (1.23–1.93)	<0.001
≥2.8	1,269	1.39 (1.08–1.79)	0.010
**Platelets**, × 10_9_**/L**			
<150	1,559	1.25 (1.03–1.53)	0.024
150–230	1,624	1.62 (1.31–2.01)	<0.001
≥230	1,672	1.59 (1.30–1.95)	<0.001
**Treatment measures**			
**Mechanical ventilation**			
Yes	1,376	1.30 (1.12–1.50)	<0.001
No	3,728	1.55 (1.30–1.84)	<0.001
**Dialysis**			
Yes	520	1.32 (0.95–1.82)	0.094
No	4,584	1.49 (1.32–1.68)	<0.001

CI, confidence interval; OR, odds ratio; SOFA, Sequential Organ Failure Assessment.

^a^Odds ratios represent the association between serum potassium level (per 1 mmol/L increase) and 28-day mortality within each stratum.

The association between serum potassium levels and 28-day mortality was evaluated using three different analytical approaches in multivariable models. Among 5,104 ICU patients with diabetes and sepsis, serum potassium levels were significantly associated with 28-day mortality. In the fully adjusted model (Model 3), each 1 mmol/L increase in serum potassium concentration was associated with a 25% higher risk of 28-day ICU mortality [OR, 1.25 (95% CI, 1.07–1.47), *P* = 0.006]. When analyzing serum potassium as a categorical variable, compared with patients with serum potassium <3.5 mmol/L, those with serum potassium >5.0 mmol/L showed significantly higher mortality risk in the unadjusted analysis [OR, 2.54 (95% CI, 1.82–3.53), *P* < 0.001]. This association remained stable after adjusting for age and sex [Model 1: OR, 2.49 (95% CI, 1.79–3.47), *P* < 0.001], slightly attenuated after additional adjustment for serum creatinine [Model 2: OR, 2.06 (95% CI, 1.46–2.92), *P* < 0.001], and remained significant although further attenuated in the fully adjusted model [Model 3: OR, 1.86 (95% CI, 1.17–2.96), *P* = 0.009]. Patients with normal potassium levels (3.5–5.0 mmol/L) showed no significant difference in mortality risk compared to those with hypokalemia across all models (Table 3). When treating serum potassium categories as ordinal variables, each category increase was associated with higher mortality risk, with the association remaining significant after full adjustment [OR, 1.38 (95% CI, 1.09–1.75); *P* = 0.008].

**Table 3:** Association of serum potassium with 28-day mortality in ICU patients with diabetes and sepsis using different analytical approaches.

	**Mortality, OR (95% CI)**			
**Analytical approach**	**Unadjusted (*****n*** = **5,104)**	**Model 1**^a^ **(*****n*** = **5,102)**	**Model 2**^b^ **(*****n*** = **5,063)**	**Model 3**^c^ **(*****n*** = **3,147)**
**Serum potassium as continuous variable**				
Per 1 mmol/L increase	1.48 (1.32–1.66)^d^	1.48 (1.32–1.66)^d^	1.37 (1.21–1.55)^d^	1.25 (1.07–1.47)^e^
**Serum potassium as categorical variable**			
<3.5 mmol/L	1 [Reference]	1 [Reference]	1 [Reference]	1 [Reference]
3.5–5.0 mmol/L	1.16 (0.87–1.54)	1.11 (0.84–1.48)	1.06 (0.79–1.42)	1.19 (0.82–1.74)
>5.0 mmol/L	2.54 (1.82–3.53)^d^	2.49 (1.79–3.47)^d^	2.06 (1.46–2.92)^d^	1.86 (1.17–2.96)^e^
**Serum potassium categories as ordinal variable** ^f^			
Per category increase	1.65 (1.38–1.97)^d^	1.64 (1.37–1.97)^d^	1.48 (1.23–1.78)^d^	1.38 (1.09–1.75)^e^

OR, odds ratio; CI, confidence interval.

^a^Model 1: Adjusted for age and sex.

^b^Model 2: Adjusted for age, sex, and serum creatinine.

^c^Model 3: Adjusted for age, sex, ethnicity, comorbidities (acute kidney injury, congestive heart failure, metastatic cancer), BMI, SOFA score, laboratory values (serum creatinine, blood urea nitrogen, glucose, serum sodium, serum chloride, albumin and platelet count), and treatment measures (mechanical ventilation and dialysis).

^d^P <0.001.

^e^P <0.05.

^f^Categories ordered as <3.5, 3.5–5.0, >5.0 mmol/L.

Data are presented as OR (95% CI). SI conversion factor: To convert potassium to mEq/L, multiply by 1.0.

## Discussion

In this large multicenter cohort study of 5,104 ICU patients with both diabetes and sepsis, we found a significant association between elevated serum potassium levels and increased 28-day mortality. Unlike the previously reported U-shaped relationship in general ICU populations (5–7), our study revealed a linear relationship between serum potassium levels and mortality risk in this specific patient population. After comprehensive adjustment for potential confounders, each 1 mmol/L increase in serum potassium was associated with a 25% higher risk of 28-day ICU mortality, and patients with hyperkalemia (>5.0 mmol/L) showed a 86% higher 28-day ICU mortality risk compared to those with hypokalemia (<3.5 mmol/L).

Our findings both confirm and extend previous research on the relationship between serum potassium and mortality in critically ill patients. While earlier studies have suggested a U-shaped relationship between potassium levels and mortality in general ICU populations (5–7), our results demonstrate a different pattern in diabetic patients with sepsis. This discrepancy might be explained by the unique pathophysiological characteristics of our study population. Notably, our findings align with previous research (11, 17, 23), which found that even mild hyperkalemia was associated with increased mortality in patients with diabetes, although these study were not specific to sepsis.

Several mechanisms might explain the observed association between hyperkalemia and increased mortality in our study population. First, diabetes and sepsis can synergistically impair potassium homeostasis through multiple pathways (24). Insulin resistance in diabetic patients can reduce cellular potassium uptake, while sepsis-induced AKI may impair potassium excretion (25, 26). Second, hyperkalemia may serve as a marker of more severe organ dysfunction (1), particularly given the higher SOFA scores observed in our hyperkalemic group. Third, the direct cardiotoxic effects of hyperkalemia may be amplified in diabetic patients, who often have underlying cardiovascular disease (4, 20, 21, 27).

Our findings have several important clinical implications. First, we suggest that the traditional U-shaped relationship between potassium and mortality may not apply to ICU patients with diabetes and sepsis, warranting a different approach to potassium management in this population. Notably, serum potassium levels exceeding 5.0 mmol/L-a commonly accepted clinical threshold-were associated with a significantly increased risk of mortality, underscoring the need for close monitoring and proactive management of hyperkalemia in ICU patients with both diabetes and sepsis. Second, the linear relationship between serum potassium levels and mortality suggests that even modest elevations in serum potassium should prompt careful clinical attention. Third, our subgroup analyses identify particularly vulnerable populations (elderly, or patients with severe organ dysfunction) who may benefit from more intensive potassium monitoring and management.

Our study has several strengths, including its large sample size, multicenter design, and comprehensive adjustment for confounders. The consistency of findings across multiple analytical approaches and subgroups supports the robustness of our results.

However, several limitations should be acknowledged. First, as an observational, retrospective study, we cannot establish causality between hyperkalemia and mortality. Second, an important limitation is the absence of time-varying analysis of potassium concentrations. Serum potassium is a dynamic parameter in the ICU setting, and our single-point measurement at admission may not capture the full exposure to dyskalemia-related risk. Repeated measurements and analysis of potassium trajectories (e.g., mean, peak, or variability) could add significant depth to understanding the association with mortality, as suggested by previous studies (7, 28, 29). This limitation was primarily due to data availability constraints in the eICU database. Third, we lacked information about pre-admission medications that might affect potassium homeostasis.

## Conclusion

In this large multicenter cohort study, we found that elevated serum potassium levels were independently associated with increased 28-day mortality among ICU patients with diabetes and sepsis. Unlike the U-shaped relationship previously observed in general ICU populations, our findings revealed a linear association between potassium levels and mortality risk in this specific patient group. These results suggest that careful monitoring and avoiding hyperkalemia may be particularly important in ICU patients with diabetes and sepsis, and that traditional thresholds for serum potassium management may need to be reconsidered for this population. Future prospective studies are needed to validate these findings and evaluate whether targeted potassium management strategies can improve outcomes in this vulnerable patient group.

## Data Availability

The original contributions presented in the study are included in the article/supplementary material, further inquiries can be directed to the corresponding authors.
